# The behavioural epidemiology of sedentary behaviour in inflammatory arthritis: where are we, and where do we need to go?

**DOI:** 10.1093/rap/rkac097

**Published:** 2023-01-24

**Authors:** Sally A M Fenton, Ciara M O’Brien, George D Kitas, Joan L Duda, Jet J C S Veldhuijzen van Zanten, George S Metsios

**Affiliations:** School of Sport, Exercise and Rehabilitation Sciences, University of Birmingham, Edgbaston, Birmingham, UK; Department of Rheumatology, Russells Hall Hospital, The Dudley Group NHS Foundation Trust, Dudley, UK; School of Psychology, Faculty of Health and Medical Sciences, University of Surrey, Surrey, UK; School of Sport, Exercise and Rehabilitation Sciences, University of Birmingham, Edgbaston, Birmingham, UK; Department of Rheumatology, Russells Hall Hospital, The Dudley Group NHS Foundation Trust, Dudley, UK; School of Sport, Exercise and Rehabilitation Sciences, University of Birmingham, Edgbaston, Birmingham, UK; School of Sport, Exercise and Rehabilitation Sciences, University of Birmingham, Edgbaston, Birmingham, UK; Department of Rheumatology, Russells Hall Hospital, The Dudley Group NHS Foundation Trust, Dudley, UK; Department of Rheumatology, Russells Hall Hospital, The Dudley Group NHS Foundation Trust, Dudley, UK; Department of Nutrition and Dietetics, School of Physical Education, Sport Science and Dietetics, University of Thessaly, Volos, Greece

**Keywords:** sedentary behaviour, sitting, behavioural epidemiology, inflammatory arthritis, intervention

## Abstract

In the last decade, studies into sedentary behaviour in inflammatory arthritis have raised important questions regarding its role in this condition. Specifically, evidence is needed on whether sedentary behaviour might exacerbate adverse inflammatory arthritis outcomes, and whether reducing sedentary behaviour might offer an effective avenue for self-management in this population. Research exploring these important research questions is still very much in its infancy and lacks the direction and scientific rigour required to inform effective intervention design, delivery and evaluation. Behavioural epidemiology refers to research that aims explicitly to understand and influence health behaviour patterns to prevent disease and improve health. To this end, the Behavioural Epidemiology Framework specifies a focused approach to health behaviour research, which leads to the development of evidence-based interventions directed at specific populations. In this review, we introduce the Behavioural Epidemiology Framework in the context of research into sedentary behaviour in inflammatory arthritis and ask: where are we, and where do we need to go?

Key messages

- Research into sedentary behaviour in inflammatory arthritis is dominated by cross-sectional studies, using heterogeneous methodologies.
- The Behavioural Epidemiology Framework outlines a sequential approach to research, to inform effective intervention design.
- More studies on sedentary behaviour in inflammatory arthritis should use experimental designs, validate measures and explore determinants.

## Introduction

Prospective observational evidence from the general population suggests that high levels of sedentary behaviour (waking activities in a seated or reclining posture, requiring ≤1.5 metabolic equivalents) [1] are linked to increased risk for all-cause and cardiovascular mortality, some cancers, and to increased incidence of type 2 diabetes and heart disease [2, 3]. This is especially the case for individuals who are not achieving recommended levels of moderate-to-vigorous physical activity (MVPA; activity ≥3 metabolic equivalents) [4, 5]. An accumulating body of experimental evidence also suggests that the pattern in which sedentary time is accumulated might have implications for cardiovascular and cardiometabolic health [6–8]. Specifically, prolonged and uninterrupted periods of sedentary time (sedentary bouts, e.g. ≥30 minutes of continuous sitting) are linked to poorer outcomes. Conversely, frequently breaking up sedentary time (sedentary breaks, e.g. with standing or light physical activity every 30 minutes) is associated with better outcomes [7, 9, 10].

Drawing from existing evidence, international guidelines now outline the importance of reducing sedentary time for health. Importantly, the most recent message is that health benefits can be achieved through increasing engagement in any intensity of physical activity, including both light physical activity (1.6–2.9 metabolic equivalents) and MVPA [2]. In essence, the underlying message advocated by health organizations across the world is to ‘move more’ [11–13]. The recommendation to ‘move more’ offers some important opportunities for encouraging meaningful, health-enhancing physical activity behaviour change. This is particularly true for clinical populations, who can find being physically active (and in particular, MVPA) a challenge.

A movement profile of both high sedentary behaviour and low MVPA is highly prevalent among people living with inflammatory arthritis [14–17]. When considering these movement behaviours, much of the focus in inflammatory arthritis has been on understanding the benefits of MVPA, in the form of structured exercise [18]. For example, a basic search of the scientific literature in March 2022 (via PubMed), returns nearly 11 000 results for ‘exercise and inflammatory arthritis’. Conversely, the terms ‘sedentary time and inflammatory arthritis’ retrieve only ∼300 articles. Nevertheless, although the evidence for the benefits of MVPA in inflammatory arthritis is unequivocal [e.g. linked to improved symptoms, lower disease activity, reduced cardiovascular disease (CVD) risk and fewer hospital admissions], systematic reviews suggest that uptake of and sustained adherence to MVPA and exercise interventions in inflammatory arthritis is problematic [18, 19]. Common barriers to MVPA and exercise in inflammatory arthritis include compromised physical function, symptoms (e.g. pain and fatigue) and fear of disease progression [20, 21]. As such, it might be the case that people living with inflammatory arthritis are more likely to engage with interventions that aim to support them to reduce their sedentary behaviour by ‘moving more’ and increasing their overall physical activity.

Based on the aforementioned epidemiological evidence, it could also be argued that not only might ‘moving more’ be more achievable than MVPA, but people living with inflammatory arthritis might stand to gain considerable health benefits by adopting interventions that aim to reduce sedentary time [22, 23]. However, this assumption is based on research conducted in the general population, and existing findings cannot be generalized to people living with inflammatory arthritis.

At present, research examining the role of sedentary behaviour in inflammatory arthritis is very much in its infancy. The majority of existing studies in this domain have used cross-sectional designs to examine associations between sedentary behaviour and various inflammatory arthritis outcomes, including inflammatory arthritis symptoms (e.g. pain and fatigue), clinical markers of disease activity and associated co-morbidities, such as CVD [22, 24]. More recent studies have begun to test interventions targeting sedentary behaviour in inflammatory arthritis, providing initial insight into the potential value of reducing sedentary behaviour for health in this patient group [25, 26].

 However, there still remain several gaps in our understanding, and there is a lack of causal, experimental research to inform effective intervention approaches. For example, we currently do not know which outcomes are likely to change in response to reducing sedentary behaviour, nor the ideal amounts and patterns of sedentary behaviour (and physical activity) in relationship to these outcomes. The potential physiological mechanisms through which sedentary behaviour might act in inflammatory arthritis are also unknown. It could be the case that the disease aetiology of inflammatory arthritis significantly impacts the physiological mechanisms hypothesized to explain the link between sedentary behaviour and increased risk of disease and mortality in non-inflammatory arthritis populations (e.g. inflammatory pathways, haemodynamic and atherosclerotic processes) [27, 28]. Accordingly, further investigations are required to develop our understanding of exactly how sedentary behaviour might be relevant in inflammatory arthritis (how much, which inflammatory arthritis outcomes are impacted, and the cause and effect mechanisms). From a psychological standpoint, we also lack knowledge regarding the specific (and modifiable) determinants of sedentary time, which is pivotal to our understanding of how we can support sedentary behaviour change effectively. Together, the information garnered via these research avenues will be crucial in developing targeted recommendations and interventions with true potential to improve inflammatory arthritis outcomes.

**Figure 1.**
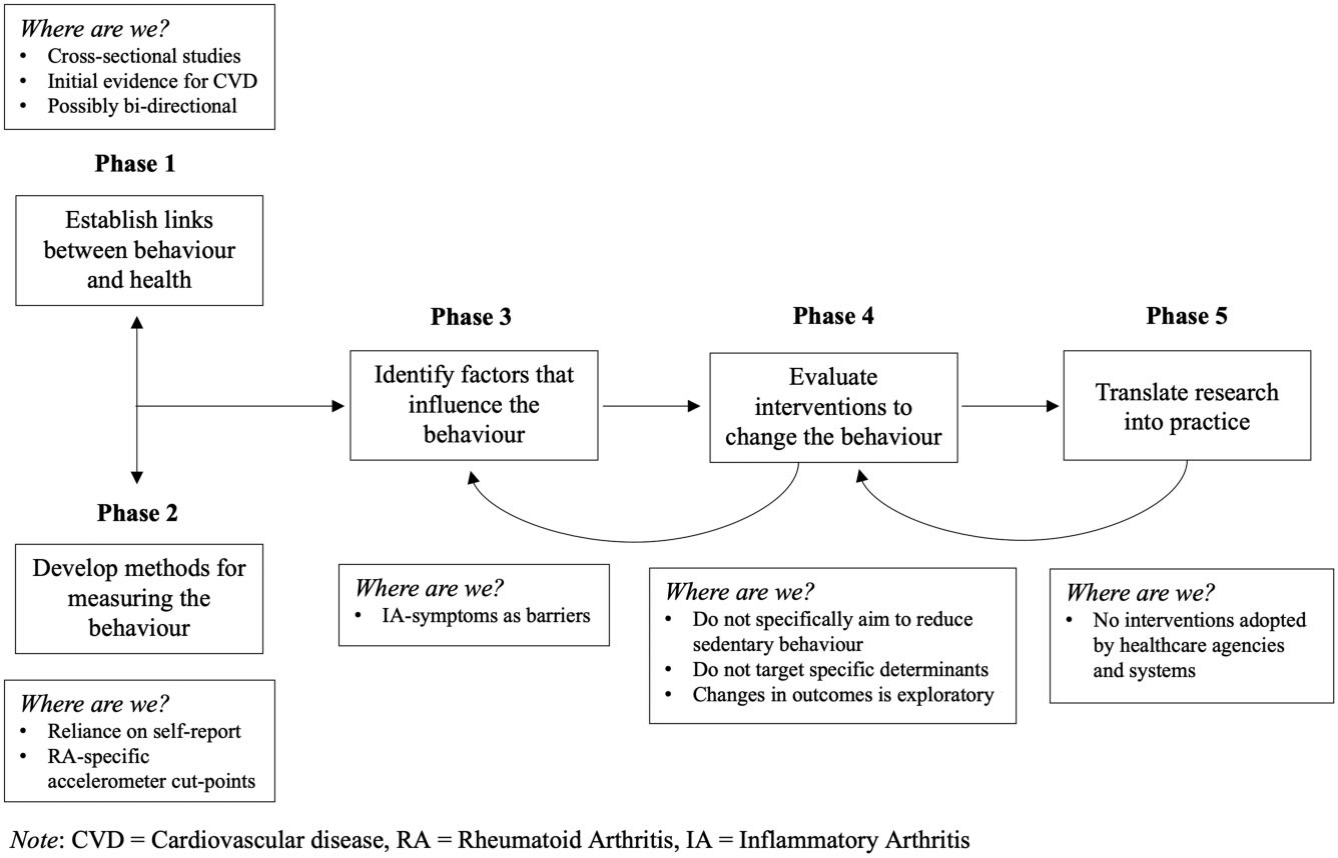
The Behavioural Epidemiology Framework in the context current of sedentary behaviour research among people living with inflammatory arthritis

A systematic approach to research on sedentary behaviour in inflammatory arthritis is required to bring this evidence together in a meaningful way, and to inform evidence-based interventions. The Behavioural Epidemiology Framework offers one such approach, setting out several research phases that facilitate identification of knowledge gaps in the evidence base that are critical to address prior to intervention development and evaluation [29]. These phases concern: (1) establishing links between behaviour and health, (2) measurement of the behaviour, (3) identifying factors influencing the behaviour (determinants), (4) interventions and (5) translation into practice (Fig. 1). Although somewhat linear, the relationships between phases are reciprocal and overlap, such that: (1) research evidence on measurement informs investigations into the links between behaviour and health, and (2) data from interventions (informed by research on determinants) can feed back to tell us more about the salience of the targeted determinants. What is crucial, is that research in each phase is conducted in the population of interest (e.g. inflammatory arthritis). As a result, where interventions are informed by methodical research evidence adhering to this framework, we can have confidence that they consider the unique characteristics (e.g. physiology and psychology) of that population. Consequently, they are likely to have greater potential to demonstrate success in promoting meaningful behaviour change (i.e. of clinical relevance).

In this review, each phase of the Behavioural Epidemiology Framework is discussed in relationship to research on sedentary behaviour in inflammatory arthritis. Key research findings in each phase are highlighted to elucidate where we are and ‘where we need to go’, with the aim of guiding researchers to develop rigorous, evidence-based interventions targeting sedentary behaviour in inflammatory arthritis.

## Phase 1: links between behaviour and health

The Behavioural Epidemiology Framework advocates that interventions should be based on evidence from population-specific research that demonstrates a link between the targeted behaviour (e.g. sedentary behaviour) and health outcomes [29]. Where interventions are guided by this knowledge, they are more likely to lead to meaningful behaviour change (i.e. change in the targeted behaviour is expected to lead to change in the targeted outcome). Developing interventions based on only an assumption that the target behaviour and health outcomes are associated (e.g. owing to findings generalized from other relevant populations) can prove futile. For example, if there is no evidence to suggest that reducing sedentary behaviour improves pain in inflammatory arthritis, on what grounds can we advocate for developing an intervention with this aim?

### Where are we?

Most current sedentary behaviour research in inflammatory arthritis has been conducted in people living with rheumatoid arthritis (RA). The majority of this research is summarized in a review published in 2018, which describes studies demonstrating links between sedentary behaviour and RA disease activity, functional disability, muscle density, bone mass and CVD risk [22]. Since 2018, research in RA has evolved to investigate a broader array of outcomes (e.g. pain, fatigue), suggesting that sedentary behaviour overall is linked to poorer physical and psychological health in people living with RA [30–32].

Beyond RA, relatively less research has examined the role of sedentary behaviour in other types of inflammatory arthritis, such as systemic lupus erythematosus (SLE), ankylosing spondylitis (AS) or psoriatic arthritis (PsA). Overall, the limited available evidence has largely focused on assessing levels and patterns of sedentary behaviour in SLE, AS and sjogren’s syndrome (SS) [14, 17, 33, 34]. A few studies have also examined associations between sedentary behaviour and indicators of CVD, sleep, physical function, quality of life and disease activity. Specifically, two studies have revealed sedentary behaviour to be linked to higher overall CVD risk scores [35] and arterial stiffness [36] in SLE. One study has reported higher sedentary behaviour to be associated with markers of sleep dysfunction in people living with SLE [37], and in AS, higher sedentary behaviour has been observed to be related to lower physical function and quality of life [16], in addition to higher disease activity [38].

### Where do we need to go?

Taken as a collective body of evidence, research into sedentary behaviour in inflammatory arthritis is only beginning. Even in RA, studies examining links between sedentary behaviour and aforementioned outcomes are typically limited in number (e.g. one or two studies per outcome) and still marked by several methodological shortcomings and inconsistencies, namely regarding a reliance on self-reported measures of sedentary behaviour (see phase 2, measurement), small samples and cross-sectional study designs [22]. Although this makes it difficult to draw definitive conclusions regarding the implications of sedentary behaviour for these conditions, research evidence leans towards the suggestion that sedentary behaviour might contribute to poorer health in inflammatory arthritis. However, carefully designed, sufficiently powered, prospective and experimental research is crucial to confirm the extent to which sedentary behaviour might impact different inflammatory arthritis outcomes.

Prospective studies with large samples and using validated measures of sedentary behaviour, will provide some initial insight into what happens to inflammatory arthritis outcomes when we observe changes in sedentary behaviour. Prospective studies also enable exploration of the interdependence between sedentary behaviour and other behaviours within the movement continuum (e.g. sleep, light physical activity and MVPA) to better understand how these behaviours relate to one another, and their potential independent and combined associations with inflammatory arthritis outcomes. For example, isotemporal substitution or compositional data analysis can be used to explore the extent to which theoretically replacing sedentary behaviour with another movement behaviour might be associated with changes inflammatory arthritis health indicators.

A recent prospective study in RA examined how changes in sedentary time over 6 months, was associated with changes in pain and fatigue. Although this study did not employ the aforementioned analytical approaches (e.g. isotemporal substitution), sedentary time was measured using a validated device (the activPAL, PAL Technologies, Glasgow, UK), and advanced statistical modelling (path analysis) was used to examine whether the relationships between sedentary time and both pain and fatigue were bi-directional. The results revealed that changes in sedentary time demonstrated a significant positive association with changes in pain and fatigue (i.e. more sedentary time is associated with more pain and fatigue) and that these associations were reciprocal, suggesting that sedentary time might represent both a cause and a consequence of pain and fatigue in RA [30]. Such findings highlight the importance of conducting hypothesis-driven experimental research, via which the potential causal role of sedentary behaviour in these relationships can be better established.

Laboratory-based experimental studies examining the acute physiological responses to sedentary behaviour will address this need and provide important insight into the mechanisms underlying the links between sedentary behaviour and different inflammatory arthritis outcomes. To date, several mechanisms have been proposed to underlie the adverse relationship between sedentary behaviour and health in non-inflammatory arthritis research [28], including impaired vascular function [39, 40] and decreased lipoprotein lipase activity (and clearance of triglycerides) [27, 41, 42], both of which might provoke deleterious reciprocal associations with systemic inflammation. Discussion of such mechanisms is beyond the scope of this review, but ultimately, experimental evidence suggests that adverse changes to these biological pathways can be attributed to the absence of skeletal muscle contraction during sedentary behaviour [27, 28].

Research examining the specific pathophysiological pathways through which sedentary behaviour might influence inflammatory arthritis outcomes is yet to be conducted. Such work will contribute an important piece of the puzzle regarding the role played by sedentary behaviour in this patient group. Laboratory-based mechanistic research will also be vital in advancing our understanding of the amounts of change/reduction in sedentary behaviour that will be likely to result in changes in inflammatory arthritis outcomes, and whether this is independent of other health-related factors (e.g. age, sex, disease activity and adiposity) and levels of physical activity. The latter is especially important; research examining sedentary behaviour mechanisms in the context of interdependent physical activity behaviours is crucial to inform effective intervention design [28]. For example, examining the physiological responses that occur when sedentary time is reduced via different patterns of physical activity (e.g. replaced by, or broken up by standing *vs* light physical activity *vs* MVPA) and whether mechanistic pathways differ or overlap, will be vital in determining whether ‘moving more’ is sufficient to improve outcomes in inflammatory arthritis.

To date, one laboratory-based experimental study has sought to address this knowledge gap, and exemplifies ‘where we need to go’ with research addressing the first phase of the Behavioural Epidemiology Framework. Pinto *et al.* [43] compared the acute effects of prolonged sitting *vs* active breaks in sitting *vs* moderate-to-vigorous exercise on cardiometabolic risk markers in RA. In this cross-over study, 15 women with RA underwent three 8-h experimental conditions: prolonged sitting (SIT); a 30-minute bout of moderate-to-vigorous exercise followed by prolonged sitting (EXERCISE); and 3-minute bouts of light-intensity walking every 30 minutes, to break up sitting (SEDENTARY BREAKS). Their results revealed that glucose, insulin and C-peptide postprandial responses were attenuated in the SEDENTARY BREAKS condition compared with the SIT condition. In addition, inflammatory cytokine [Interleukin-1β (IL-1β) and Tumour Necrosis Factor-alpha (TNF-α)] concentrations decreased during the SEDENTARY BREAKS conditions, compared with increases seen during EXERCISE. The authors concluded that brief active breaks in sitting with light-intensity activity might offset markers of cardiometabolic disturbance. The findings of Pinto *et al.* [43] provide the first evidence that replacing sedentary time with periods of light physical activity (and ‘moving more’) might produce meaningful changes in important inflammatory arthritis outcomes. This research also aligns with the body of evidence to suggest that sedentary time might play a particularly important role in CVD risk for this population [15, 31, 44].

Findings from laboratory-based experimental research will provide crucial knowledge to ensure that longer-term free-living sedentary behaviour change interventions are designed with greater potential to lead to clinically meaningful changes in inflammatory arthritis outcomes. Specifically, mechanistic findings, such as those outlined above, can be used to inform the selection of inflammatory arthritis outcomes in free-living interventions. This is based on the premise that over time (e.g. ≥12 weeks), long-term reductions in daily sedentary behaviour might culminate in longer-lasting changes in the clinical end-points related to the identified mechanisms (e.g. endothelial dysfunction causing atherosclerosis and related cardiovascular co-morbidity). Indeed, free-living interventions will provide crucial insight into the impact of sustained changes in sedentary behaviour over time for people with inflammatory arthritis, and shed light on the potential clinical efficacy of different approaches. For example, a free-living intervention based on the study by Pinto *et al.* [43] could encourage people with RA to break up their sitting every 30 minutes with light-intensity physical activities. Outcomes would include biomarkers of cardiometabolic health.

## Phase 2: measurement of the target behaviour

The Behavioural Epidemiology Framework specifies a reciprocal relationship between phase 1 (links) and phase 2 (measurement), such that knowledge regarding accurate measurement of sedentary behaviour is crucial to inform research into links between sedentary behaviour and health, and vice versa; that is, insight into specific sedentary behaviour patterns/domains linked to health in inflammatory arthritis, can inform more targeted research into validation of measures that can assess these patterns and domains more accurately.

Current measurement techniques in sedentary behaviour research are split broadly into self-report and device-based measures. Self-report methods encompass questionnaires (e.g. international physical activity questionnaire (IPAQ) [45]) and diaries (e.g. Bouchard physical activity record [46]). Device-based measures include accelerometers and posture sensors, which afford the ability to monitor free-living sedentary time continuously through changes in body accelerations or posture.

### Where are we?

Questionnaires have been used most frequently to measure sedentary behaviour in inflammatory arthritis in research to date, perhaps owing to their ease of application and relatively low cost and participant burden [47]. However, questionnaires are subject to social desirability bias and inaccuracies in participant recall, and have been criticized owing to the tendency of participants to under-report levels of sedentary behaviour [48]. For example, Yu *et al.* [49] used Bland–Altman analysis to compare the agreement between IPAQ *vs* accelerometer-assessed sedentary time in people with RA. The authors discovered that patients underestimated sedentary time when responding to the IPAQ, when compared with the study criterion of accelerometry.

Relative to questionnaires, accelerometers offer a more objective approach to measurement of sedentary time, and devices such as the Actigraph (Actigraph, Florida, USA) and GENEActiv (Activinsights, Cambridgeshire, UK) are being used increasingly in studies of inflammatory arthritis [14, 50–52]. Accelerometers work by capturing raw acceleration data that are analysed to quantify sedentary time on the basis of low acceleration/movement. Currently, the majority of scientists use research-grade accelerometers and rely on the manufacturer’s software and proprietary algorithms (e.g. Actilife and Activinsights) to reduce the complexities of processing large volumes of raw accelerometer data [53, 54]. In general, software provided by manufacturers works by compressing raw data to generate a metric termed ‘activity-counts’. Validated thresholds or ‘cut-points’ (typically developed using the criterion of indirect calorimetry) can then be applied to these activity-counts to define periods of sedentary time and physical activity spent at different intensities [55, 56]. A common cut-point used to define sedentary time is <100 activity-counts per minute [57, 58].

From the early 2000s, studies started to use accelerometers to measure physical activity in arthritic populations, with an exponential increase seen over the last decade [14, 17, 22, 59]. Interest directed toward sedentary behaviour in inflammatory arthritis emerged only ∼6–7 years ago, and until recently, accelerometers had not been validated specifically for measurement of sedentary time (or physical activity) in people living with inflammatory arthritis [22]. Instead, researchers have largely relied on algorithms built into the manufacturer’s software to analyse their data (e.g. activity-count based accelerometer cut-points), which have been developed in validation studies of healthy adults [56, 57]. This is particularly problematic when we consider that the physiology and associated activity patterns of people living with inflammatory arthritis are likely to differ substantially from those among healthy adults in the general population. For example, relative to non-inflammatory arthritis populations, the higher basal metabolic rate characteristic of inflammatory arthritis means that a lower accelerometer cut-point is likely to correspond to MVPA in this patient group [60]. Therefore, the current ‘one size fits all’ approach to measurement of free-living sedentary behaviour might have resulted in inaccurate estimates of sedentary time, impacting the precision of existing research into sedentary behaviour in inflammatory arthritis [61].

### Where do we need to go?

To make progress in this field, inflammatory arthritis-specific analytical approaches to sedentary time (and physical activity) measurement are required. Studies in RA are again leading the way in this regard, with researchers beginning to validate device-based measures of sedentary time in this population [58, 62]. In regards to accelerometry, O'Brien *et al.* [58] recently published the first study to validate a popular research-grade accelerometer in people living with RA. In their study, the Actigraph GT3X+ accelerometer was validated against indirect calorimetry to develop RA-specific triaxial accelerometer activity-count based cut-points for measuring sedentary time, light- and moderate-intensity physical activity in RA [58]. In the same study, a field-based validation protocol examined the validity of the RA-specific triaxial sedentary time cut-point, compared with the widely used non-RA uniaxial sedentary time cut-point of <100 counts per minute [57]. The results revealed that the RA-specific cut-point was a more valid alternative to the non-RA sedentary time cut-point, highlighting the need to validate accelerometers in other inflammatory arthritis populations to ensure more accurate measurement of sedentary time in these patient groups.

Although the study by O’Brien *et al.* [58] offers an encouraging move towards the adoption of validated, inflammatory arthritis-specific measurement methods, there are still some important analytical considerations to highlight. First, while no other inflammatory arthritis-specific accelerometer cut-points are available, inflammatory arthritis researchers are limited to applying either those developed for people living with RA (i.e. O’Brien *et al.* [58]) or those developed in non-inflammatory arthritis populations. Second, even where inflammatory arthritis-specific accelerometer cut-points are developed, in the absence of expertise in computer science (or related fields), researchers are still likely to require manufacturer’s software (and proprietary algorithms) to analyse their activity-count based data. Although this can offer a somewhat simplified approach to analysis, the algorithms used to calculate activity-counts are often protected and vary across device manufacturers. This lack of standardization with regard to measurement protocols (i.e. different devices and different software) and analytical techniques (i.e. different, non-inflammatory arthritis specific cut-points) introduces bias into accelerometer data processing, making accurate comparisons across studies in inflammatory arthritis challenging [55, 61, 63].

The issues highlighted above are also common in research conducted in other populations and patient groups [56, 61, 64]. To address this challenge, world-leading physical activity and sedentary behaviour researchers are advocating a move towards analytical approaches that use raw accelerometer data (milli-gs, *mg*) rather than algorithms developed by manufacturers [55]. Indeed, the collection and analysis of accelerometer data saved as raw signals, rather than proprietary accelerometer activity-counts, enables transparent and replicable data-transformation methods that can be carried out after data-processing. This approach will facilitate comparison between accelerometer outputs across studies, regardless of which brand of device was used (e.g. Actigraph or GENEActiv), and will lead to improved measurement precision and generalizability of recommendations for sedentary behaviour in inflammatory arthritis [65]. However, the use of raw acceleration data also presents a new and different challenge, whereby without the simplicity of proprietary algorithms, the researcher is now responsible for processing and analysing huge amounts of data. Consequently, where raw acceleration data are to be used in the context of sedentary behaviour research in inflammatory arthritis, expertise from researchers with backgrounds in mathematics, computer science, engineering and statistics is likely to be of crucial importance.

Still, whilst accelerometers offer significant opportunity to facilitate progress in the field of sedentary behaviour in inflammatory arthritis, they do not offer a perfect measurement solution. Accelerometers quantify sedentary time on the basis of a lack of movement/acceleration, rather than posture (i.e. whether a person is sitting or lying), which is an important facet of the definition of sedentary behaviour (i.e. activity ≤1.5 metabolic equivalents and a sitting/reclining/lying posture) [1]. In this way, the activPAL^TM^ offers an advance over accelerometers for free-living assessment of sedentary time and is currently considered the gold standard to measure sedentary time in field-based research [66].

The activPAL^TM^ is a small, lightweight device, typically worn attached to the front of the thigh, that uses proprietary algorithms to classify free-living behaviour as sitting/lying (sedentary), based on posture and acceleration [66]. The activPAL^TM^ is also able to measure breaks in sedentary time (i.e. where sitting is broken up by standing or ambulatory activity). To date, two studies have validated the activPAL^TM^ against direct observation in the RA population, reporting high classification accuracy (98%) and strong agreement between activPAL^TM^-assessed sedentary time and sedentary breaks with direct observation [58, 62]. Three studies in RA have also used the activPAL^TM^ to investigate the role of sedentary time in inflammatory arthritis: two exploring the cross-sectional or longitudinal associations between sedentary time with inflammatory arthritis outcomes (RA and AS), and one randomized controlled trial examining changes in sedentary time in response to intervention [16, 25, 30, 67].

Based on the above, it would seem prudent to suggest that recommending the activPAL^TM^ as the measure of choice for sedentary behaviour research in inflammatory arthritis is ‘where we need to go’. However, the activPAL^TM^ has its own limitations. First, the activPAL^TM^ does not provide a measure of the intensity of physical activity. Capturing data on physical activity in synchrony with sedentary time is crucial to answer questions regarding interrelationships between these behaviours, aiding our understanding of how amounts and patterns of sedentary behaviour are linked to inflammatory arthritis outcomes [28]. Second, a global limitation of device-based measures (i.e. the activPAL and accelerometers) is that they can only assess sedentary time, rather than a specific behaviour *per se*. This is particularly important when trying to understand the role of specific types of sedentary behaviours for health in inflammatory arthritis. For example, when exploring the link between sedentary behaviour and fatigue or wellbeing in inflammatory arthritis, it would be important to differentiate between sedentary behaviours that are cognitively stimulating and involve positive social interaction, compared to those that are perhaps more passive and undertaken alone [68].

Bearing this in mind, it seems that there is currently no single perfect solution to the measurement of sedentary behaviour in inflammatory arthritis. However, advances in technology and artificial intelligence are soon likely to offer novel, comprehensive approaches for measurement of sedentary time, which could be validated for use in inflammatory arthritis. For example, machine learning is being used to develop classification algorithms able to measure volumes, patterns and types of sedentary behaviours (and physical activities) from raw accelerometer data in non-inflammatory arthritis populations [69]. However, in the short term, and in the absence of validated machine learning approaches, it might be appropriate for researchers to use both self-report and device-based measures of sedentary behaviour in order to capture the amount, patterns and context of free-living sedentary time.

## Phase 3: identify factors that influence the behaviour

Research shows that behaviour change interventions are likely to be more effective when the factors (determinants) that influence the specific behaviour of interest (e.g. sedentary behaviour) have been identified and targeted [70]. Determinants offer a basis for intervention development by representing the mechanisms of action for an intervention (i.e. if the determinant is impacted/changed, this is assumed to lead to behavioural change) and. In addition, determinants can be used to inform intervention content and strategies (e.g. the selection of evidence-based behaviour change techniques likely to have a positive impact on the determinant) [71]. Research conducted under phase 3 of the Behavioural Epidemiology Framework therefore has the primary purpose of identifying relevant determinants, and providing empirical and/or theoretical evidence to demonstrate that they are linked to the target behaviour.

### Where are we?

Existing research exploring the determinants of sedentary behaviour in inflammatory arthritis is largely comprised of quantitative, cross-sectional studies, exploring the role of symptoms (e.g. pain, fatigue and physical function) as determinants (barriers) to sedentary behaviour [16, 30, 32, 33, 37]. Most of this research has been conducted in the RA population and has been highlighted in the sections above (i.e. phase 1); that is, owing to: (1)the cross-sectional study designs that dominate inflammatory arthritis research to date, and (2) the likely bi-directional relationship between inflammatory arthritis symptoms and sedentary behaviour, studies exploring links between sedentary behaviour and health in inflammatory arthritis (i.e. phase 1) could also be argued to represent research into determinants of sedentary behaviour.

### Where do we need to go?

Research that explores the complex (and potentially reciprocal) relationships between sedentary behaviour and inflammatory arthritis symptoms is crucial to understanding how we can effectively support people living with inflammatory arthritis to reduce their sedentary behaviour. Indeed, symptom-related barriers to (i.e. determinants) sedentary behaviour change need to be understood and properly addressed in interventions if they are to be successful. For example, we previously highlighted a prospective observational study reporting bi-directional relationships between pain and fatigue with sedentary time in RA [30]. Based on these findings, we would suggest that sedentary behaviour interventions are designed to include behaviour change approaches to address pain as a barrier [e.g. adopt ‘if–then’ planning (‘if’ I experience pain, ‘then’ I will…)], and/or, to support changes in sedentary behaviour at times when the experience of pain is less severe and/or disease activity is well controlled.

In parallel, studies that seek to identify the relative salience of other more changeable determinants, will be crucial to inform sedentary behaviour interventions with the potential to overcome some of the symptom-related barriers. Research operating from a socio-ecological perspective, considering other malleable determinants, will be instrumental in this regard [72]. For example, studies exploring factors influencing sedentary behaviour in inflammatory arthritis at the individual (e.g. health, wellbeing and psychological factors), environmental (e.g. home, work, social/cultural, built *vs* natural environment) and organizational (e.g. politics, economics and health-care context) levels, in addition to the interrelationships between these factors, are likely to provide a detailed and comprehensive landscape upon which to develop interventions. Unpicking the relative salience of each determinant and its impact on sedentary time (and interdependent physical activity behaviours) requires carefully designed research able to explore the dynamics of these factors in depth.

Future studies in this domain should be carefully planned and adopt suitable methodological designs. Owing to the lack of current evidence in this area, initial work should be exploratory and adopt a bottom-up, inductive approach. First, qualitative research should investigate individual, environmental and organizational level factors that influence daily sedentary behaviour among people living with inflammatory arthritis (e.g. via interviews and focus groups). Qualitative studies can explore the in-depth lived experiences of individuals and groups, and have huge potential to uncover the complex and interrelated determinants of sedentary behaviour in inflammatory arthritis. To date, however, only one qualitative study has sought specifically to investigate determinants of sedentary behaviour in inflammatory arthritis [73, 74]. Through semi-structured interviews, Thomsen *et al.* [74] revealed that people living with RA engage in sedentary behaviours, such as reading, doing crossword puzzles or watching television, ‘when symptoms dominate’ (e.g. pain and fatigue). Significantly more qualitative research in inflammatory arthritis is required to develop our understanding of the multi-level, dynamic factors that influence sedentary behaviour in these patient groups. Subsequently, findings from qualitative studies can be tested in quantitative longitudinal, proof-of-concept research, prior to intervention.

**Figure 2.**
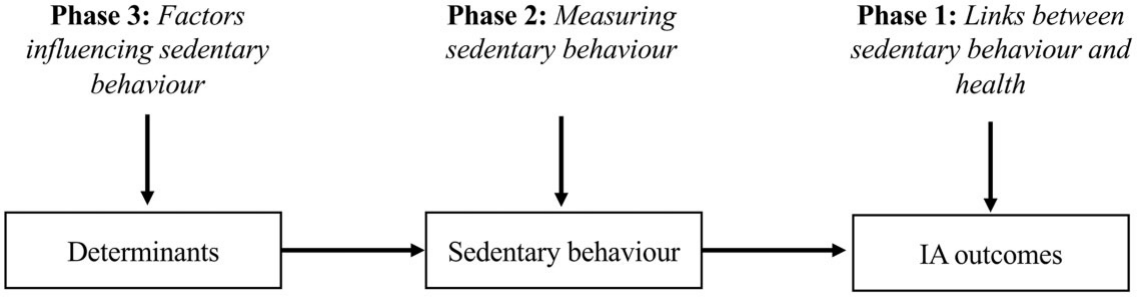
Illustration of how longitudinal studies informed by phases 1, 2 and 3 of the Behavioural Epidemiology Framework can inform intervention design. IA: Inflammatory arthritis

Importantly, where these longitudinal studies are informed by phases 1 and 2 of the Behavioural Epidemiology Framework, they can elucidate the extent to which interventions targeting specific determinants might have the potential to encourage meaningful sedentary behaviour change; for example, studies that examine how changes in identified determinants of sedentary behaviour are related to changes in (volumes and patterns of) sedentary behaviour and, in turn, changes in inflammatory arthritis outcomes. This approach offers a dose–response ‘process’ or ‘logic’ model, which will provide some indication of the extent of sedentary behaviour change achieved by targeting a particular determinant, and how this degree of change relates to downstream changes in pertinent inflammatory arthritis outcomes (Fig. 2).

In a similar vein, research informed by psychological theories of behaviour change (e.g. self-efficacy theory [75] and self-determination theory [76]) will be vital in revealing ‘what works’ when it comes to supporting sedentary behaviour change in inflammatory arthritis [71]. Indeed, psychological theories can provide a systematic framework to inform the selection and development of intervention strategies (i.e. based on the assumption that they will positively impact the identified psychological determinant) and specify the psychological processes assumed to result in behaviour change [71]. When proof-of-concept research can successfully bring together phases 1–3 of the framework, and is grounded in psychological theory, we will gain considerable insight into ‘how things work’, from both a psychological standpoint (i.e. the psychosocial processes supporting behaviour change) and a physiological standpoint (i.e. how much particular inflammatory arthritis outcomes are impacted, cause and effect, mechanisms), before intervention [71].

## Phase 4: intervention

In phase 4 (interventions), knowledge generated from phases 1, 2 and 3 of the Behavioural Epidemiology Framework is bought together to inform intervention design and evaluation. This ensures that interventions to support sedentary behaviour change are designed to:

1. target inflammatory arthritis outcomes that have demonstrated proven links with sedentary behaviour in experimental research (phase 1), and,
2. that the former has been achieved by encouraging changes in the amounts and patterns of sedentary behaviour in a manner shown to influence the responsible pathophysiological mechanisms (phase 1).
3. measure changes in sedentary behaviour using validated, inflammatory arthritis-specific methods, and employ a combination of self-report and device-based methods (phase 2).
4. address the specific determinants of sedentary behaviour for people living with inflammatory arthritis, considering the fact that some inflammatory arthritis outcomes might represent both causes (barriers) and consequences of sedentary behaviour (phase 3).

These requirements are crucial to ensure that interventions are designed with the rigour needed to successfully promote changes in sedentary behaviour (i.e. targeting determinants, owing to phase 3) in the manner and to the extent necessary (i.e. volume, bouts, breaks, owing to phase 2), to be able to evaluate the clinical efficacy of interventions for inflammatory arthritis (i.e. do they promote meaningful behaviour change?).

### Where are we?

Current interventions that have aimed to reduce sedentary behaviour in inflammatory arthritis have been developed using evidence from only select phases of the framework; that is, they were not hypothesis driven regarding the inflammatory arthritis outcomes assessed (i.e. not informed by framework phase 1) and/or did not base their intervention approach on research into the determinants of sedentary behaviour in inflammatory arthritis (i.e. not informed by framework phase 3). The extent to which valid measurement approaches were used to assess changes in sedentary behaviour was variable (i.e. phase 2). Considering the time and resource required to design, deliver and evaluate interventions, existing research likely represents missed opportunities to truly explore the potential efficacy of these intervention approaches, to advance understanding in this domain.

For example, although a recent physical activity intervention for patients with RA and SLE aimed to reduce sedentary behaviour, this was included as a secondary objective [77]. The effects of the intervention on RA and SLE outcomes were also exploratory. As a result, the intervention, assessments and outcomes were not designed with the intention of definitively testing the role of sedentary behaviour for any particular outcome in these patient groups. Results revealed no change in accelerometer-assessed sedentary behaviour (using non-inflammatory arthritis specific cut-points) after the intervention, whereas significant improvements in pain were reported. This raises important questions; for example, does this mean that sedentary behaviour is not important for pain in inflammatory arthritis, or merely that the study lacked direction and scientific rigour to determine the role of sedentary behaviour in this regard? Although intervention research conducted in this manner may indeed reveal some interesting findings, this is likely to be more by chance than intent.

To illustrate further, a recent randomized controlled trial examined the effectiveness of a behaviour change intervention (motivational counselling and SMS text reminders) to reduce sedentary behaviour (total sitting time) in people with RA [25]. However, the study was not designed or powered to detect changes in RA outcomes in response to the intervention. Therefore, whilst the intervention demonstrated reductions in sedentary time and parallel improvements in some RA outcomes, the extent to which the reductions in sedentary time were responsible for the observed improvements in RA outcomes could not be deduced. In addition, the researchers did not control for other potential factors that might explain variability in RA outcomes (e.g. adiposity and medication), and the interdependence with physical activity was not considered when evaluating changes in RA outcomes; that is, it is possible that increases in physical activity resulting from the reduction in sedentary behaviour contributed to some of the changes in the outcomes that were observed. The authors suggested that study participants replaced their sedentary time with standing, but it is also likely that participants increased their overall movement and light-intensity physical activity (i.e. they were ‘moving more’). However, we are left questioning specifically how variation in the patterns of standing and light physical activity between patients impacted the RA outcomes assessed, and asking what were the physiological mechanisms operating?

### Where do we need to go?

The intervention studies by Li *et al.* [77]) and Thomsen *et al.* [25] underline the need for a more targeted, systematic approach to interventional research in sedentary behaviour in inflammatory arthritis, which is offered by the Behavioural Epidemiology Framework. Interventions developed using this framework will be crucial in determining how to change sedentary behaviour successfully, and how changes in targeted volumes and patterns of sedentary behaviour translate to meaningful changes in specific inflammatory arthritis outcomes. Where possible, future interventions should aim to target longer-term changes in sedentary behaviour (e.g. 6–12 months) that extend beyond the typical time line of 2–3 months often observed in lifestyle research. Although this is particularly challenging from a behaviour change standpoint, longer-term interventions will be vital in establishing the impact that sustained reductions sedentary behaviour might have on inflammatory arthritis-specific outcomes and overall health. The study by Thomsen *et al.* [25, 26], has provided some insight into the acceptability of longer-term sedentary behaviour interventions in RA. They demonstrated that participants in the intervention group were still significantly less sedentary and reported more favourable RA outcomes (e.g. lower visual analog scale pain and fatigue) than control group participants at the 18-month follow-up [26]. However (and as stated above), the design of this intervention means that the direct effects of reducing sedentary behaviour on RA outcomes cannot be determined (e.g. cofounders and interdependent activity behaviours were not controlled for, and no dose–response ‘process’ or ‘logic model’ was examined).

At this point, based on the limited evidence available, it is only possible to suggest that interventions focused on ‘moving more’, and on breaking up sedentary time with light physical activity, might provoke beneficial changes in factors related to cardiometabolic and CVD risk in inflammatory arthritis [15, 25, 78]. Given that CVD is the leading cause of death among people with inflammatory arthritis [79], it might be prudent to pursue research aligned to phases 1 and 2 of the framework, with these foci. With regard to phase 3, initial research suggests that self-determination theory might offer a useful framework to inform interventions to reduce sedentary behaviour in RA [71, 80]. Indeed, recent research revealed that autonomous (self-determined) motivation might be an important determinant of sedentary time in RA, which could be explored in more detail.

## Phase 5: translate research into practice

Research in phase 5 of the Behavioural Epidemiology Framework should evaluate and describe how to disseminate, adopt and implement effective sedentary behaviour change interventions (i.e. supported in phase 4) successfully across different settings. Phase 5 might also inspire new research in phases 1–4 of the Behavioural Epidemiology Framework [e.g. barriers to implementation might be considered in phase 3 (determinants) research].

### Where are we?

To date, no sedentary behaviour change interventions in inflammatory arthritis have been adopted by health-care agencies or systems. This state of affairs might be expected, owing to the paucity of research evidence in this field.

### Where do we need to go?

Studies addressing phases 1–4 of the Behavioural Epidemiology Framework in the development of sedentary behaviour change interventions in inflammatory arthritis should consistently offer recommendations for translating research findings into practice [81]. For example, the use of comparative-effectiveness designs in research studies across phases 1–4, such as observational research, randomized controlled trials and systematic reviews, can inform health-care decisions by identifying the most effective intervention for an individual’s needs, abilities and motivations [82] (i.e. what works best for who, and how?). Comparative-effectiveness designs can be employed to compare: (1) the health outcomes of interventions targeting different components of the movement continuum (e.g. sedentary behaviour *vs* MVPA), (2) the health outcomes of interventions targeting different volumes, bouts, breaks and types of sedentary behaviour, and (3) the socio-ecological setting of interventions aiming to reduce sedentary behaviour (e.g. environmental level *vs* individual level), in inflammatory arthritis [72]. Furthermore, it is essential to consider the cost, reach, potential adverse effects and sustainability of an intervention [83].

It is also important to evaluate the health-promotion messages associated with sedentary behaviour change, to optimally communicate and raise awareness of the benefits of reducing sedentary behaviours in inflammatory arthritis. For example, the message ‘sit less’ is not very inclusive in this population (i.e. some people with inflammatory arthritis are wheelchair users); therefore, changing the language in the message to ‘move more’ might be more acceptable. Research that examines the efficacy and acceptability of such health-promotion messages would offer a valuable avenue for research aligned with phase 5 of the Behavioural Epidemiology Framework.

Engaging and working with key stakeholders within health-care systems and multidisciplinary teams is also vital to the dissemination, implementation, adoption and maintenance of sedentary behaviour change interventions in inflammatory arthritis [81, 83]. Improving implementation intelligence in these areas can be achieved by organizing qualitative research and advisory groups, which can stimulate new research and thus feedback to earlier phases of the Behavioural Epidemiology Framework. Translating research in a clinical population, such as inflammatory arthritis, to practice within health-care systems seems to be the most appropriate place to start. However, people living with inflammatory arthritis also engage with built environments, communities and work environments, among other contexts. These settings should not be ignored.

## Conclusion

Interventions targeting sedentary behaviour might have significant potential to improve health among people living with inflammatory arthritis. However, existing research into the implications of sedentary behaviour in inflammatory arthritis is somewhat sporadic, and lacks the direction and scientific rigour required to inform effective intervention design (‘where we are’). The Behavioural Epidemiology Framework offers a systematic methodology to direct research into sedentary behaviour in inflammatory arthritis, and outlines a sequential approach to conducting research across the spectrum of descriptive, explanatory, analytical and intervention studies. We therefore recommend that researchers should conduct studies aligned with the Behavioural Epidemiology Framework (Box 1), with a particular focus on acute laboratory-based studies (phase 1, to explore outcomes and responsible physiological mechanisms), validation of device-based measures of sedentary behaviour, including exploration of new approaches (e.g. machine learning, phase 2) and theory-based determinants research aligned with socio-ecological models (i.e. considering individual, organizational and environmental factors, phase 3). As research within phases 1–3 of the framework accumulates, it will be crucial to triangulate data sources to inform proof-of-concept studies. These studies will be instrumental in directing the design, delivery and evaluation of effective interventions to reduce sedentary behaviour in inflammatory arthritis (i.e. ‘where we need to go’).

Box 1. Sedentary behaviour in inflammatory arthritis: where we need to go, according to the Behavioural Epidemiology Framework

**Phase 1:** research using prospective and experimental study designs

- Prospective studies with large samples, using validated measures of sedentary behaviour and advanced statistical modelling techniques (e.g. isotemporal substitution and compositional data analysis)
- Laboratory-based experimental studies to establish potential mechanisms underlying the links between sedentary behaviour and different inflammatory arthritis outcomes, and whether these differ when sedentary time is reduced/replaced via different patterns of physical activity

**Phase 2:** research employing methods that have been validated specifically for measurement of sedentary behaviour among people living with inflammatory arthritis

- Studies should use a combination of self-report and device-based measurement methods (e.g. accelerometers, the activPAL^TM^) until more advanced analytical approaches are available (i.e. machine learning of raw accelerometer data to quantify volume, patterns and types of sedentary behaviours in inflammatory arthritis)
- Where possible, device-based methods should enable the analysis of raw accelerometer data, to which existing data-transformation methods (e.g. cut-points) can be applied post data-processing
- Where proprietary algorithms are used (e.g. activity-counts), these should be validated and calibrated in inflammatory arthritis (e.g. inflammatory arthritis specific cut-points, see [58] for example in RA).

**Phase 3:** qualitative and quantitative research taking a bottom-up, inductive approach

- Qualitative research should explore the complex individual-, environmental- and organizational-level factors that influence daily sedentary behaviour among people living with inflammatory arthritis, and how these factors are interrelated
- Determinants identified in qualitative research should inform quantitative longitudinal proof-of-concept studies. These studies should examine how changes in identified determinants are related to changes in (volumes and patterns) of sedentary behaviour and, in turn, inflammatory arthritis outcomes (i.e. to determine dose–response ‘logic’ models)
- Both quantitative and qualitative research should be grounded in psychological theories of behaviour change, in order to understand ‘how things work’ from a psychological standpoint

**Phase 4:** interventions developed and evaluated using knowledge generated from phases 1, 2 and 3 of the Behavioural Epidemiology Framework

- Shorter-term interventions (2–3 months) to provide initial insight into the potential acceptability and health impacts of reducing sedentary behaviour in inflammatory arthritis
- As the field progresses, longer-term interventions will become increasingly important to gain a better understanding of how sustained (e.g. 6–12 months) changes in sedentary behaviour might improve inflammatory arthritis specific outcomes and overall health

**Phase 5:** research to generate knowledge supporting the adoption and implementation of effective sedentary behaviour change interventions in different settings. This could include:

- Comparative-effectiveness designs to compare the relative efficacy of sedentary behaviour interventions conducted in different contexts (e.g. environmental level *vs* individual level) for improving health outcomes in inflammatory arthritis
- Research that examines the efficacy and acceptability of health-promotion messages about reducing sedentary behaviour
- Research engaging key stakeholders within health-care systems, communities and occupational contexts, in order to improve implementation intelligence

## Data Availability

All data retrieved for this article were collated via a search of published papers in the relevant literature. As such, all data used to write the article are available in published scientific articles cited in this publication.

## References

[1] Tremblay MS , Aubert S, Barnes JD et al; on behalf of SBRN Terminology Consensus Project Participants. Sedentary Behavior Research Network (SBRN) – Terminology Consensus Project process and outcome. Int J Behav Nutr Phys Act 2017;14:75. 28599680 10.1186/s12966-017-0525-8 PMC5466781

[2] Ekelund U , Tarp J, Steene-Johannessen J et al Dose-response associations between accelerometry measured physical activity and sedentary time and all cause mortality: systematic review and harmonised meta-analysis. BMJ 2019;366:l4570. 31434697 10.1136/bmj.l4570 PMC6699591

[3] de Rezende LFM , Rodrigues Lopes M, Rey-López JP, Matsudo VKR, Luiz ODC. Sedentary behavior and health outcomes: an overview of systematic reviews. PLoS One 2014;9:e105620. 25144686 10.1371/journal.pone.0105620 PMC4140795

[4] Ekelund U , Tarp J, Fagerland MW et al Joint associations of accelerometer-measured physical activity and sedentary time with all-cause mortality: a harmonised meta-analysis in more than 44 000 middle-aged and older individuals. Br J Sports Med 2020;54:1499–506. 33239356 10.1136/bjsports-2020-103270 PMC7719907

[5] Ekelund U , Brown WJ, Steene-Johannessen J et al Do the associations of sedentary behaviour with cardiovascular disease mortality and cancer mortality differ by physical activity level? A systematic review and harmonised meta-analysis of data from 850 060 participants. Br J Sports Med 2019;53:886–94. 29991570 10.1136/bjsports-2017-098963

[6] Diaz KM , Howard VJ, Hutto B et al Patterns of sedentary behavior and mortality in U.S. middle-aged and older adults: a national cohort study. Ann Intern Med 2017;167:465–75. 28892811 10.7326/M17-0212 PMC5961729

[7] Bellettiere J , Winkler EAH, Chastin SFM et al Associations of sitting accumulation patterns with cardio-metabolic risk biomarkers in Australian adults. PLoS One 2017;12:e0180119. 28662164 10.1371/journal.pone.0180119 PMC5491133

[8] Saunders TJ , Larouche R, Colley RC, Tremblay MS. Acute sedentary behaviour and markers of cardiometabolic risk: a systematic review of intervention studies. J Nutr Metab 2012;2012:712435. 22754695 10.1155/2012/712435 PMC3382951

[9] Saunders TJ , Atkinson HF, Burr J et al The acute metabolic and vascular impact of interrupting prolonged sitting: a systematic review and meta-analysis. Sports Med 2018;48:2347–66. 30078066 10.1007/s40279-018-0963-8

[10] Chastin SF , Egerton T, Leask C, Stamatakis E. Meta-analysis of the relationship between breaks in sedentary behavior and cardiometabolic health. Obesity (Silver Spring) 2015;23:1800–10. 26308477 10.1002/oby.21180

[11] Powell KE , King AC, Buchner DM et al The scientific foundation for the physical activity guidelines for Americans, 2nd edition. J Phys Act Health 2019;16:1–11. 10.1123/jpah.2018-0618 30558473

[12] Ross R , Chaput J-P, Giangregorio LM et al Canadian 24-hour movement guidelines for adults aged 18–64 years and adults aged 65 years or older: an integration of physical activity, sedentary behaviour, and sleep. Appl Physiol Nutr Metab 2020;45:S57–102. 33054332 10.1139/apnm-2020-0467

[13] Department of Health and Social Care. Chief Medical Officers’ Physical Activity Guidelines. 2019. https://assets.publishing.service.gov.uk/government/uploads/system/uploads/attachment_data/file/832868/uk-chief-medical-officers-physical-activity-guidelines.pdf (4 April 2022, date last accessed).

[14] Legge A , Blanchard C, Hanly JG. Physical activity and sedentary behavior in patients with systemic lupus erythematosus and rheumatoid arthritis. Open Access Rheumatol 2017;9:191–200. 29184453 10.2147/OARRR.S148376 PMC5687492

[15] Fenton SAM , Veldhuijzen van Zanten JJCS, Kitas GD et al Sedentary behaviour is associated with increased long-term cardiovascular risk in patients with rheumatoid arthritis independently of moderate-to-vigorous physical activity. BMC Musculoskelet Disord 2017;18:131. 28356089 10.1186/s12891-017-1473-9 PMC5404687

[16] Coulter EH , McDonald MT, Cameron S, Siebert S, Paul L. Physical activity and sedentary behaviour and their associations with clinical measures in axial spondyloarthritis. Rheumatol Int 2020;40:375–81. 31848736 10.1007/s00296-019-04494-3 PMC7002460

[17] van Genderen S , Boonen A, van der Heijde D et al Accelerometer quantification of physical activity and activity patterns in patients with ankylosing spondylitis and population controls. J Rheumatol 2015;42:2369–75. 26523021 10.3899/jrheum.150015

[18] Rausch Osthoff AK , Juhl CB, Knittle K et al Effects of exercise and physical activity promotion: meta-analysis informing the 2018 EULAR recommendations for physical activity in people with rheumatoid arthritis, spondyloarthritis and hip/knee osteoarthritis. RMD Open 2018;4:e000713. 30622734 10.1136/rmdopen-2018-000713 PMC6307596

[19] Rausch Osthoff AK , Niedermann K, Braun J et al 2018 EULAR recommendations for physical activity in people with inflammatory arthritis and osteoarthritis. Ann Rheum Dis 2018;77:1251–60. 29997112 10.1136/annrheumdis-2018-213585

[20] Veldhuijzen van Zanten JJCS , Rouse PC, Hale ED et al Perceived barriers, facilitators and benefits for regular physical activity and exercise in patients with rheumatoid arthritis: a review of the literature. Sports Med 2015;45:1401–12. 26219268 10.1007/s40279-015-0363-2 PMC4579262

[21] Passalent L , Cyr A, Jurisica I et al Motivators, barriers, and opportunity for e-health to encourage physical activity in axial spondyloarthritis: a qualitative descriptive study. Arthritis Care Res (Hoboken) 2022;74:50–8. 34928533 10.1002/acr.24788

[22] Fenton SAM , Veldhuijzen van Zanten JJCS, Duda JL, Metsios GS, Kitas GD. Sedentary behaviour in rheumatoid arthritis: definitions, measurement and implications for health. Rheumatology (Oxford) 2018;57:213–26. 28398519 10.1093/rheumatology/kex053

[23] Fenton SAM , Kitas GD. Rheumatoid arthritis: sedentary behaviour in RA – a new research agenda. Nat Rev Rheumatol 2016;12:698–700. 27811913 10.1038/nrrheum.2016.179

[24] Pinto AJ , Roschel H, de Sa Pinto AL et al Physical inactivity and sedentary behavior: overlooked risk factors in autoimmune rheumatic diseases? Autoimmun Rev 2017;16:667–74. 28479487 10.1016/j.autrev.2017.05.001

[25] Thomsen T , Aadahl M, Beyer N et al The efficacy of motivational counselling and SMS reminders on daily sitting time in patients with rheumatoid arthritis: a randomised controlled trial. Ann Rheum Dis 2017;76:1603–6. 28584189 10.1136/annrheumdis-2016-210953 PMC5561370

[26] Thomsen T , Aadahl M, Beyer N et al Sustained long-term efficacy of motivational counseling and text message reminders on daily sitting time in patients with rheumatoid arthritis: long-term follow-up of a randomized, parallel-group trial. Arthritis Care Res 2020;72:1560–70. 10.1002/acr.24060 31507095

[27] Hamilton MT , Healy GN, Dunstan DW, Zderic TW, Owen N. Too little exercise and too much sitting: inactivity physiology and the need for new recommendations on sedentary behavior. Curr Cardiovasc Risk Rep 2008;2:292–8. 22905272 10.1007/s12170-008-0054-8 PMC3419586

[28] Dempsey PC , Matthews CE, Dashti SG et al Sedentary behavior and chronic disease: mechanisms and future directions. J Phys Act Health 2020;17:52–61. 31794961 10.1123/jpah.2019-0377

[29] Sallis JF , Owen N, Fotheringham MJ. Behavioral epidemiology: a systematic framework to classify phases of research on health promotion and disease prevention. Ann Behav Med 2000;22:294–8. 11253440 10.1007/BF02895665

[30] O'Brien CM , Ntoumanis N, Duda JL et al Pain and fatigue are longitudinally and bi-directionally associated with more sedentary time and less standing time in rheumatoid arthritis. Rheumatology (Oxford) 2021;60:4548–57. 33493311 10.1093/rheumatology/keab029 PMC8487306

[31] Hammam N , Ezeugwu VE, Rumsey DG, Manns PJ, Pritchard-Wiart L. Physical activity, sedentary behavior, and long-term cardiovascular risk in individuals with rheumatoid arthritis. Phys Sportsmed 2019;47:463–70. 31122104 10.1080/00913847.2019.1623995

[32] O'Leary H , Larkin L, Murphy GM, Quinn K. Relationship between pain and sedentary behavior in rheumatoid arthritis patients: a cross-sectional study. Arthritis Care Res (Hoboken) 2021;73:990–7. 32277738 10.1002/acr.24207

[33] Margiotta DPE , Basta F, Dolcini G et al Physical activity and sedentary behavior in patients with systemic lupus erythematosus. PLoS One 2018;13:e0193728. 29505598 10.1371/journal.pone.0193728 PMC5837187

[34] Ng WF , Miller A, Bowman SJ et al; UK Primary Sjögren’s Syndrome Registry. Physical activity but not sedentary activity is reduced in primary Sjogren's syndrome. Rheumatol Int 2017;37:623–31. 28013357 10.1007/s00296-016-3637-6 PMC5357288

[35] Legge A , Blanchard C, Hanly JG. Physical activity, sedentary behaviour and their associations with cardiovascular risk in systemic lupus erythematosus. Rheumatology (Oxford) 2020;59:1128–36. 31691832 10.1093/rheumatology/kez429

[36] Morillas-de-Laguno P , Vargas-Hitos JA, Rosales-Castillo A et al Association of objectively measured physical activity and sedentary time with arterial stiffness in women with systemic lupus erythematosus with mild disease activity. PLoS One 2018;13:e0196111. 29694382 10.1371/journal.pone.0196111 PMC5919022

[37] Margiotta DPE , Laudisio A, Navarini L et al Pattern of sleep dysfunction in systemic lupus erythematosus: a cluster analysis. Clin Rheumatol 2019;38:1561–70. 30693395 10.1007/s10067-018-04410-3

[38] O'Dwyer T , O'Shea F, Wilson F. Decreased physical activity and cardiorespiratory fitness in adults with ankylosing spondylitis: a cross-sectional controlled study. Rheumatol Int 2015;35:1863–72. 26254884 10.1007/s00296-015-3339-5

[39] Taylor FC , Pinto AJ, Maniar N, Dunstan DW, Green DJ. The acute effects of prolonged uninterrupted sitting on vascular function: a systematic review and meta-analysis. Med Sci Sports Exerc 2022;54:67–76. 34334722 10.1249/MSS.0000000000002763

[40] da Silva GO , Santini LB, Farah BQ et al Effects of breaking up prolonged sitting on cardiovascular parameters: a systematic review. Int J Sports Med 2022;43:97–106. 34535019 10.1055/a-1502-6787

[41] Hamilton MT , Hamilton DG, Zderic TW. Exercise physiology versus inactivity physiology: an essential concept for understanding lipoprotein lipase regulation. Exerc Sport Sci Rev 2004;32:161–6. 15604935 10.1097/00003677-200410000-00007 PMC4312662

[42] Hamilton MT , Hamilton DG, Zderic TW. Role of low energy expenditure and sitting in obesity, metabolic syndrome, type 2 diabetes, and cardiovascular disease. Diabetes 2007;56:2655–67. 17827399 10.2337/db07-0882

[43] Pinto AJ , Meireles K, Pecanha T et al Acute cardiometabolic effects of brief active breaks in sitting for patients with rheumatoid arthritis. Am J Physiol Endocrinol Metab 2021;321:E782–94. 34693756 10.1152/ajpendo.00259.2021

[44] Fenton SAM , Ntoumanis N, Duda JL et al Diurnal patterns of sedentary time in rheumatoid arthritis: associations with cardiovascular disease risk. RMD Open 2020;6:e001216. 32669453 10.1136/rmdopen-2020-001216 PMC7425187

[45] Craig CL , Marshall AL, Sjostrom M et al International physical activity questionnaire: 12-country reliability and validity. Med Sci Sports Exerc 2003;35:1381–95. 12900694 10.1249/01.MSS.0000078924.61453.FB

[46] Bouchard C , Tremblay A, Leblanc C et al A method to assess energy expenditure in children and adults. Am J Clin Nutr 1983;37:461–7. 6829488 10.1093/ajcn/37.3.461

[47] Atkin AJ , Gorely T, Clemes SA et al Methods of measurement in epidemiology: sedentary behaviour. Int J Epidemiol 2012;41:1460–71. 23045206 10.1093/ije/dys118 PMC3465769

[48] Chastin SFM , Dontje ML, Skelton DA et al; Seniors USP Team. Systematic comparative validation of self-report measures of sedentary time against an objective measure of postural sitting (activPAL). Int J Behav Nutr Phys Act 2018;15:21. 29482617 10.1186/s12966-018-0652-x PMC5828279

[49] Yu CA , Rouse PC, Veldhuijzen Van Zanten JJ et al Subjective and objective levels of physical activity and their association with cardiorespiratory fitness in rheumatoid arthritis patients. Arthritis Res Ther 2015;17:59. 25885649 10.1186/s13075-015-0584-7 PMC4384324

[50] O'Brien CM , Duda JL, Kitas GD et al Correlates of sedentary behaviour and light physical activity in people living with rheumatoid arthritis: protocol for a longitudinal study. Mediterr J Rheumatol 2018;29:106–17. 32185311 10.31138/mjr.29.2.106 PMC7046072

[51] Summers G , Booth A, Brooke-Wavell K, Barami T, Clemes S. Physical activity and sedentary behavior in women with rheumatoid arthritis: a comparison of patients with low and high disease activity and healthy controls. Open Access Rheumatol 2019;11:133–42. 31417323 10.2147/OARRR.S203511 PMC6592056

[52] Dassouki T , Benatti FB, Pinto AJ et al Objectively measured physical activity and its influence on physical capacity and clinical parameters in patients with primary Sjogren's syndrome. Lupus 2017;26:690–7. 27798360 10.1177/0961203316674819

[53] Rowlands AV. Moving forward with accelerometer-assessed physical activity: two strategies to ensure meaningful, interpretable, and comparable measures. Pediatr Exerc Sci 2018;30:450–6. 30304982 10.1123/pes.2018-0201

[54] Rowlands AV , Mirkes EM, Yates T et al Accelerometer-assessed physical activity in epidemiology: are monitors equivalent? Med Sci Sports Exerc 2018;50:257–65. 28976493 10.1249/MSS.0000000000001435

[55] Rowlands AV , Edwardson CL, Davies MJ et al Beyond cut points: accelerometer metrics that capture the physical activity profile. Med Sci Sports Exerc 2018;50:1323–32. 29360664 10.1249/MSS.0000000000001561

[56] Gorman E , Hanson HM, Yang PH et al Accelerometry analysis of physical activity and sedentary behavior in older adults: a systematic review and data analysis. Eur Rev Aging Phys Act 2014;11:35–49. 24765212 10.1007/s11556-013-0132-x PMC3990855

[57] Troiano RP , Berrigan D, Dodd KW et al Physical activity in the United States measured by accelerometer. Med Sci Sports Exerc 2008;40:181–8. 18091006 10.1249/mss.0b013e31815a51b3

[58] O'Brien CM , Duda JL, Kitas GD et al Measurement of sedentary time and physical activity in rheumatoid arthritis: an ActiGraph and activPAL validation study. Rheumatol Int 2020;40:1509–18. 32472303 10.1007/s00296-020-04608-2 PMC7371657

[59] Negrini F , de Sire A, Lazzarini SG et al Reliability of activity monitors for physical activity assessment in patients with musculoskeletal disorders: a systematic review. J Back Musculoskelet Rehabil 2021;34:915–23. 33935067 10.3233/BMR-200348

[60] Roubenoff R , Roubenoff RA, Cannon JG et al Rheumatoid cachexia: cytokine-driven hypermetabolism accompanying reduced body cell mass in chronic inflammation. J Clin Invest 1994;93:2379–86. 8200971 10.1172/JCI117244 PMC294444

[61] Pedisic Z , Bauman A. Accelerometer-based measures in physical activity surveillance: current practices and issues. Br J Sports Med 2015;49:219–23. 25370153 10.1136/bjsports-2013-093407

[62] Larkin L , Nordgren B, Purtill H et al Criterion validity of the activPAL activity monitor for sedentary and physical activity patterns in people who have rheumatoid arthritis. Phys Ther 2016;96:1093–101. 26637646 10.2522/ptj.20150281

[63] Fenton SAM , Veldhuijzen van Zanten J, Duda JL, Metsios GS, Kitas GD. Sedentary behaviour in rheumatoid arthritis: definition, measurement and implications for health. Rheumatology (Oxford) 2018;57:213–26. 28398519 10.1093/rheumatology/kex053

[64] Bianchim MS , McNarry MA, Larun L et al Calibration and validation of accelerometry using cut-points to assess physical activity in paediatric clinical groups: a systematic review. Prev Med Rep 2020;19:101142. 32637301 10.1016/j.pmedr.2020.101142 PMC7327836

[65] Rowlands AV , Fairclough SJ, Yates T et al Activity intensity, volume, and norms: utility and interpretation of accelerometer metrics. Med Sci Sports Exerc 2019;51:2410–22. 31318713 10.1249/MSS.0000000000002047

[66] Edwardson CL , Winkler EAH, Bodicoat DH et al Considerations when using the activPAL monitor in field-based research with adult populations. J Sport Health Sci 2017;6:162–78. 30356601 10.1016/j.jshs.2016.02.002 PMC6188993

[67] Thomsen T , Aadahl M, Beyer N et al Motivational counselling and SMS-reminders for reduction of daily sitting time in patients with rheumatoid arthritis: a descriptive randomised controlled feasibility study. BMC Musculoskelet Disord 2016;17:434. 27756265 10.1186/s12891-016-1266-6 PMC5070122

[68] Copeland JL , Ashe MC, Biddle SJ et al Sedentary time in older adults: a critical review of measurement, associations with health, and interventions. Br J Sports Med 2017;51:1539. 28724714 10.1136/bjsports-2016-097210

[69] Willetts M , Hollowell S, Aslett L, Holmes C, Doherty A. Statistical machine learning of sleep and physical activity phenotypes from sensor data in 96,220 UK Biobank participants. Sci Rep 2018;8:7961. 29784928 10.1038/s41598-018-26174-1 PMC5962537

[70] Prince SA , Saunders TJ, Gresty K, Reid RD. A comparison of the effectiveness of physical activity and sedentary behaviour interventions in reducing sedentary time in adults: a systematic review and meta-analysis of controlled trials. Obes Rev 2014;15:905–19. 25112481 10.1111/obr.12215 PMC4233995

[71] Fenton SAM , Duda JL, Veldhuijzen van Zanten J, Metsios GS, Kitas GD. Theory-informed interventions to promote physical activity and reduce sedentary behaviour in rheumatoid arthritis: a critical review of the literature. Mediterr J Rheumatol 2020;31:19–41. 32411931 10.31138/mjr.31.1.19 PMC7219651

[72] O'Donoghue G , Perchoux C, Mensah K et al; DEDIPAC Consortium. A systematic review of correlates of sedentary behaviour in adults aged 18–65 years: a socio-ecological approach. BMC Public Health 2016;16:163. 26887323 10.1186/s12889-016-2841-3 PMC4756464

[73] Pearson NA , Tutton E, Martindale J et al Qualitative interview study exploring the patient experience of living with axial spondyloarthritis and fatigue: difficult, demanding and draining. BMJ Open 2022;12:e053958. 10.1136/bmjopen-2021-053958 PMC8883261 35217538

[74] Thomsen T , Beyer N, Aadahl M et al Sedentary behaviour in patients with rheumatoid arthritis: a qualitative study. Int J Qual Stud Health Well-being 2015;10:28578. 26462971 10.3402/qhw.v10.28578 PMC4604213

[75] Bandura A. Self-efficacy: toward a unifying theory of behavioral change. Psychol Rev 1977;84:191–215. 847061 10.1037//0033-295x.84.2.191

[76] Deci EL , Ryan RM. The support of autonomy and the control of behavior. J Pers Soc Psychol 1987;53:1024–37. 3320334 10.1037//0022-3514.53.6.1024

[77] Li LC , Feehan LM, Xie H et al Efficacy of a physical activity counseling program with use of a wearable tracker in people with inflammatory arthritis: a randomized controlled trial. Arthritis Care Res (Hoboken) 2020;72:1755–65. 32248626 10.1002/acr.24199

[78] Pinto AJ , Peçanha T, Meireles K et al A randomized controlled trial to reduce sedentary time in rheumatoid arthritis: protocol and rationale of the Take a STAND for Health study. Trials 2020;21:171. 32051025 10.1186/s13063-020-4104-y PMC7014778

[79] Anyfanti P , Dara A, Angeloudi E et al Monitoring and managing cardiovascular risk in immune mediated inflammatory diseases. J Inflamm Res 2021;14:6893–906. 34934338 10.2147/JIR.S276986 PMC8684400

[80] Fenton SAM , Veldhuijzen van Zanten JJCS, Metsios GS et al Testing a self-determination theory-based process model of physical activity behavior change in rheumatoid arthritis: results of a randomized controlled trial. Transl Behav Med 2021;11:369–80. 32203571 10.1093/tbm/ibaa022 PMC7963285

[81] Metsios GS , Fenton SA, Moe HR et al; IMPACT-RMD Consortium. Implementation of Physical Activity into routine Clinical pracTice in Rheumatic Musculoskeletal Disease: the IMPACT-RMD study protocol and rationale. Mediterr J Rheumatol 2019;30:231–6. 32467876 10.31138/mjr.30.4.231 PMC7241658

[82] Rosenberg DE , Lee IM, Young DR et al Novel strategies for sedentary behavior research. Med Sci Sports Exerc 2015;47:1311–5. 25222817 10.1249/MSS.0000000000000520 PMC4362872

[83] Peters DH , Adam T, Alonge O, Agyepong IA, Tran N. Implementation research: what it is and how to do it. BMJ 2013;347:f6753. 24259324 10.1136/bmj.f6753

